# Physical and Antioxidant Properties of Cassava Starch–Carboxymethyl Cellulose Incorporated with Quercetin and TBHQ as Active Food Packaging

**DOI:** 10.3390/polym12020366

**Published:** 2020-02-07

**Authors:** Wirongrong Tongdeesoontorn, Lisa J. Mauer, Sasitorn Wongruong, Pensiri Sriburi, Pornchai Rachtanapun

**Affiliations:** 1 School of Agro-Industry, Mae Fah Luang University, Chiang Rai 57100, Thailand; wirongrong.ton@mfu.ac.th; 2 Research Unit of Innovative Food Packaging and Biomaterials, Mae Fah Luang University, Chiang Rai 57100, Thailand; 3 Department of Food Science, Purdue University, West Lafayette, IN 47907, USA; mauer@purdue.edu; 4 Division of Biotechnology, Faculty of Agro-Industry Chiang Mai University, Chiang Mai 50100, Thailand; sasitorn.w@cmu.ac.th; 5 Department of Chemistry, Faculty of Science, Chiang Mai University, Chiang Mai 50200, Thailand; pensiri@scinece.cmu.ac.th; 6 Division of Packaging Technology, Faculty of Agro-Industry, Chiang Mai University, Chiang Mai 50100, Thailand; 7 Cluster of Agro Bio-Circular-Green Industry (Agro BCG), Chiang Mai University, Chiang Mai 50100, Thailand

**Keywords:** active packaging, antioxidant film, biopolymers, CMC, cassava starch and food applications

## Abstract

Antioxidant integration has been advocated for in polymer films, to exert their antioxidative effects in active packaging. In this study, the new antioxidant food packaging made from cassava starch–carboxymethyl cellulose (CMC), which is biodegradable, edible and inexpensive, was developed. Their properties were determined and applied in food models for application. Antioxidants (quercetin and tertiary butylhydroquinone (TBHQ)) were added at various concentrations into cassava starch–carboxymethyl cellulose (CMC) (7:3 *w*/*w*) films containing glycerol (30 g/100 g starch–CMC) as a plasticizer. The effects of quercetin and TBHQ concentrations on the mechanical properties, solubility, antioxidative activity, and applications of the films were investigated. Addition of antioxidant improved tensile strength, but reduced elongation at break of the cassava starch–CMC film. Cassava starch–CMC films containing quercetin showed higher tensile strength, but lower elongation at break, compared to films with TBHQ. Increases in quercetin and TBHQ content decreased water solubility in the films. Both the total phenolic content and antioxidative activity (DPPH scavenging assay) still remained in films during storage time (30 days). In application, cassava starch–CMC film containing quercetin and TBHQ can retard the oxidation of lard (35–70 days) and delay the discoloration of pork.

## 1. Introduction

The development of biodegradable films based on biopolymers has attracted attention, mainly due to their friendliness to the environment and their potential as a substitute for some petroleum polymers in the food packaging industry. Biodegradable films have generally been made of renewable, natural, and abundant biopolymeric materials, such as polysaccharides, proteins, lipids, or a combination of these components. The biodegradable films based on blends of polysaccharides–polysaccharides such as starch–methylcellulose [1], pullulan–starch [2], chitosan–starch, and chitosan–pullulan [3]; CMC-rice starch [4] has also been investigated. Depending on the interactions between components, these formulas of blended film can improve the mechanical properties and gas/moisture barrier properties of the films in some cases. 

Antioxidants are widely used as food additives to improve the oxidation stability of lipids and to prolong product shelf-life, mainly for dried products and O_2_-sensitive foods. Synthetic phenolic antioxidants (SPAs), such as butylated hydroxytoluene (BHT), butylated hydroxyanisole (BHA), and tertiary butylhydroquinone (TBHQ), are commonly used because of their chemical stability, low cost, and availability. The most suitable antioxidant for vegetable oils is TBHQ [5], because TBHQ produces greater stability than BHT and is more cost-effective [6]. However, the safety of these SPAs has been questioned. There is much interest among food manufacturers in using natural antioxidants (such as tocopherol, ascorbic acid, and quercetin) as replacements for the synthetic antioxidants currently used [7]. Antioxidants have been suggested for integration into polymer films to exert their antioxidative effect [8]. Lopez-de-Dicastillo [9] developed ethylene vinyl alcohol (EVOH)-based films containing natural antioxidants (catechin and quercetin). Therefore, there is interest in the use of plant extracts and essential oils, as antioxidants to replace synthetic additives [10,11]. This has another positive effect, as a part of the agro-industrial by-products will become an economical and practical source of potent antioxidants [11,12]. Crouvisier-Urion et al. [13] reported that the incorporation of lignin from wood extraction into chitosan film provided to the film a radical scavenging activity, essentially governed by a surface activity mechanism; and with high-pressure homogenization processing, could also increase the hydrophobicity of chitosan-lignin film, which can be useful in application [14]. Hargens-Madsen et al. [15] used tocopherols in the edible film to improve precooked meat quality. Oussalah et al. [16] added essential oils in milk protein-based film. Another natural antioxidant used in the production of films consists of extracts obtained from different varieties of tea [11]. Nisa et al. [17] produced a film with potato starch as a matrix biopolymer, and 5% green tea as an antioxidant, which was kept in direct contact with fresh beef. Similarly, Yang et al. [18] developed a film with grain proteins as matrix biopolymer and different amounts of green, black, and oolong tea extracts as antioxidants, which were applied to fresh pork. Meanwhile, Qin et al. [19] used chitosan as a matrix biopolymer, and tea polyphenols solution as natural antioxidants, to develop a film to protect cooked pork sausages against oxidation. In these studies, samples that were wrapped with active films had lower lipid oxidation ratios than the control. Besides, Nisa et al. [17] and Qin et al. [19] also reported that the application of the active films increased the stability of the red color and prevented discoloration.

However, little information on the effect of antioxidants on cassava starch-based films and their mechanical properties is available. Rachtanapun et al. [20] reported that antioxidants (propyl gallate (PG), butylhydroxyanisole (BHA), and butylhydroxytoluene (BHT)) had no effect on tensile strength, % elongation, and folding endurance of rice flour/cassava starch blended film. In the previous study, cassava starch–CMC films were successfully prepared, and their physical properties were determined [21]. Cassava films with higher concentrations of bixin nanocapsules exhibited a significant decrease in tensile strength and water solubility, an increase in elongation at break and water vapor permeability, as well as significant improvement in protection against UV and visible light, delaying the oxidation rate of sunflower oil [22]. A cassava starch bio-based active film, incorporated with aqueous green tea extract and oil palm colorant, was used to pack butter (maintained for 45 days) under accelerated oxidation conditions and showed the lower peroxide index (231.57%) [23]. 

Research on the application of antioxidant biodegradable films on food models, especially lard and fatty food, is limited. The objectives of this study were to develop new active cassava starch–CMC films, evaluate the effect of antioxidants (quercetin and TBHQ) on film physical properties, and determine the antioxidative effect of these films on lard and pork quality. 

## 2. Materials and Methods

### 2.1. Materials

Cassava starch (Bangkok Inter Food Co., LTD., Bangkok, Thailand) was used to make the films. CMC (AKUCELL-AF 0705, average molecular weight = 150,000 Daltons) was donated by Akzo Nobel (Amsterdam, The Netherlands). The 2, 2-Diphenyl-1-picryl hydrazyl (DPPH) was purchased from Fluka (Mexico City, USA). Quercetin and tertiary butylhydroquinone (TBHQ) were obtained from Alfa Aesar (Massachusetts, USA) and Fluka (Buchs, Switzerland), respectively. Folin–Ciocalteu reagent, glycerol, methanol, glacial acetic acid, and chloroform were purchased from Merck (Darnstadt, Germany). Magnesium chloride and magnesium nitrate were purchased from Ajax Finechem (New South Wales, Australia) were used to control storage relative humidity.

### 2.2. Film Preparation

The cassava starch–CMC film and antioxidant casting procedure were modified from a published method [21]. The film solution was prepared by dispersing 7 g of cassava starch and 3 g of CMC in distilled water (200 mL) with various quercetin and TBHQ contents (0, 20, 50, 100 mg). Glycerol (30 g/100 g cassava starch–CMC mixture) was used as a plasticizer. The film solution was heated to 80 °C with constant stirring to achieve starch gelatinization. The film-forming solution was then cast on a flat 30 × 30 cm Teflon plate. The films were then dried at room temperature (25 °C) for 24 h. 

### 2.3. Mechanical Properties

The blended films were cut into 25 × 100 mm strips and then conditioned in desiccators over saturated salt solutions with the desired relative humidity 34% (MgCl_2_) and 54% RH (Mg(NO_3_)_2_) at 25 °C for 48 h before testing. The mechanical properties (tensile strength and elongation at break) of the films were measured using a universal testing machine (Hounsfield, UK) according to the American Society for Testing and Materials (ASTM) D 882-12 method [24]. Twenty replicates of each film type, preconditioned at each RH, were tested.

### 2.4. Fourier Transform Infrared Spectroscopy (FT-IR) 

Transmission infrared spectra of the films were measured at room temperature using a Nicolet 6700 FT-IR spectrometer (Thermo Electron Corporation, Waltham, Massachusetts, USA) in the range of 4000–400 cm^−1^ with 64 scans, 4 cm^−1^ resolution, using a deuterated triglycine sulfate (DTGS) KBr detector and KBr beam splitter. The films were placed in the sample holder. 

### 2.5. Differential Scanning Calorimetry (DSC)

Differential scanning calorimetry (Mettler Toledo Schwerzenbach Instrument, Ohio, USA) was carried out. Samples were previously conditioned at 54% RH and 25 °C at least 48 h before testing. Three replicates of the film samples (≈10 milligrams) were put in aluminum pans and heated in the temperature range of −20 to 220 °C, at a heating rate of 5 °C/min in a nitrogen atmosphere (50 mL/min). 

### 2.6. X-ray Diffraction (XRD)

The X-ray diffraction patterns of cassava starch–CMC films with and without antioxidants were carried on an X’Pert MPD X-ray diffractometer (Philips, Amsterdam, The Netherlands), using Nickel-filtered Cu Kα radiation at 40 kV and 35 mA in the 2θ range of 5°–50°.

### 2.7. Water Solubility of Composite Films

The water solubility of composite films was measured as a percentage of dry residue after the film was soaked in water for 24 h. This method was adapted from the method by Phan, et al. [25]. The initial dry weight of each film was obtained after drying film specimens at 65 °C for 24 h, followed by placement in 0% RH silica gel desiccators for two days. Dried films (about 0.3 g) were weighed (initial dry weight) and immersed in beakers containing 50 mL distilled water at 25 °C, that were then sealed and periodically agitated for 24 h. The solutions containing film residues were filtered with Whatman filter paper No.1 (previously dried at 105 °C for 24 h and weighed before using). The residues were dried at 80 °C for 24 h and weighed to determine the weight of dry matter (final dry weight). Tests were performed in triplicate, and the solubility was calculated using Equation (1):

(1) $$\mathrm{solubility}\left(\%\right)=\left(\frac{\mathrm{initial}\;\mathrm{dried}\;\mathrm{weight}-\mathrm{final}\;\mathrm{dried}\;\mathrm{weight}\;}{\mathrm{initial}\;\mathrm{dried}\;\mathrm{weight}}\right)\times 100$$

### 2.8. Total Phenolic Assay

For this determination, 3 × 3 cm (≈0.1 g) of three-film samples were cut randomly from the big sheet of film and dissolved in 10 mL of methanol for 24 h to prepare a film extract solution. The total phenolic content (TPC) of the film samples was determined according to the Folin–Ciocalteu method as described by Association of Official Analytical Chemists (AOAC) [26] with slight modifications. Briefly, 0.5 mL of film extract solution was added with 8 mL distilled water and 1 mL of Folin–Ciocalteu reagent. The mixture was incubated for 5 min at room temperature before the addition of 0.5 mL of saturated sodium carbonate. The mixture was stored in a dark chamber at room temperature for 30 min. The absorbance of the mixture was then measured at 760 nm using a spectrophotometer (Spectro SC, Labomed Inc., Los Angeles, California, USA). Methanol was used as a blank. The concentration of total phenolic compounds in the samples is expressed as gallic acid equivalent (GAE), which reflects the phenolic content as the amount of gallic acid in mg per gram dry weight of the sample, calculated by using Equation (2) [27]:

(2) $$A_{760}=0.001\;mg\;gallic\;acid+0.027,$$

where *A*_760_ is the absorbance at 760 nm.

### 2.9. Determination of Antioxidant Activity in the Composite Films 

Film samples (3 × 3 cm, with at least three pieces randomly cut) were dissolved in 10 mL methanol for 24 h and filtered. The sample extract (500 μL) was mixed with 2 mL of 0.06 mM DPPH solution (in methanol) and kept in a dark location for 30 min at room temperature. The absorbance was then measured at 517 nm with a spectrophotometer. Methanol solution and quercetin were used as a reference and positive control, respectively. The radical scavenging activity of DPPH was calculated according to the following Equation (3):

(3) $$Radical\;scavenging\;activity=\left(\frac{A_{reference}-A_{sample}}{A_{sample}}\right)\times 100,$$

where *A* is the absorbance.

### 2.10. Application of Antioxidant Films on Lard

#### 2.10.1. Effect of Antioxidant Incorporations into Cassava Starch–CMC Films on Lard Storage 

The effect of antioxidants (quercetin and TBHQ) in the composite film on lard storage was determined using a method modified from Zhang et al. [28]. The lard samples (15 mL, 36 °C) were packaged in cassava starch–CMC-quercetin films and cassava starch–CMC-TBHQ films, with a film area of 100 cm^2^ each, and stored at 30 °C and relative humidity of 40% for 30 days. The control was unpackaged and stored under the same conditions. The peroxide value of the packaged lard was determined.

#### 2.10.2. Estimation of Peroxide Value

The peroxide values of the film extracts were measured using the modified method of Jung, et al. [29]. One gram of the film extract was dissolved in 25 mL of solvent (2 parts chloroform: 3 parts acetic acid). Saturated potassium iodide (1 mL) was then added, and the solution was kept in the dark for 10 min. After that, 30 mL of distilled water and 1 mL of starch solution (1 g/100 mL) were added to the solution and titrated with 0.01 N Na_2_S_2_O_3_ until colorless. Peroxide values (PVs) were calculated as follows (Equation (4)):

(4) $$Peroxide\;Value\;\left(PV\right)=\frac{\left(S-B\right)\times N\times 1000}{W},$$

where *S, B, N* (mol equiv/L), and W mean the titration amount of sample, the titration amount of blank, the normality of Na_2_S_2_O_3_, and the sample weight (*W*, g), respectively.

### 2.11. Application of Antioxidant Films on Fresh Pork 

The procedures in this experiment were applied from other studies [16,29,30]. Fresh pork samples were purchased from a local butcher shop, the samples were sliced into 5 × 10 × 1.5 cm (width × length × thickness) sections and weighed ca. 30–35 g. Each piece of sliced pork was placed in a polystyrene tray (10 × 5 × 1.5 cm) and covered on either side with one of the antioxidant composite films. Trays were sealed hermetically and stored at 4 ± 1 °C. Color changes of the pork were observed periodically (on days 0, 4, 8, and 12) during storage. The color characteristics were evaluated using a hand-held colorimeter (Minolta, Japan) to determine the L* value (lightness or brightness), the a* value (redness or greenness), and the b* value (yellowness or blueness) of the film samples. Percentage of redness decrease was calculated from the a* value following Equation (5):

(5) $$\%\;Redness\;decrease\;=\frac{\left(a_{0}^{*}-a_{t}^{*}\right)\times 100}{a_{0}^{*}},$$

where $a_{0}^{*}$ is the a* value of the sample at 0 days, and $a_{t}^{*}$ is the a* value at storage time.

### 2.12. Statistical Analysis

ANOVA analysis and Duncan’s multiple range tests were performed on all results using a statistical program, SPSS v. 10.0, at a confidence interval of 95% to determine the significant differences between group samples.

## 3. Results and Discussion

### 3.1. Influence of Antioxidants Concentrations on Mechanical Properties of the Composite Films

**Figure 1:**
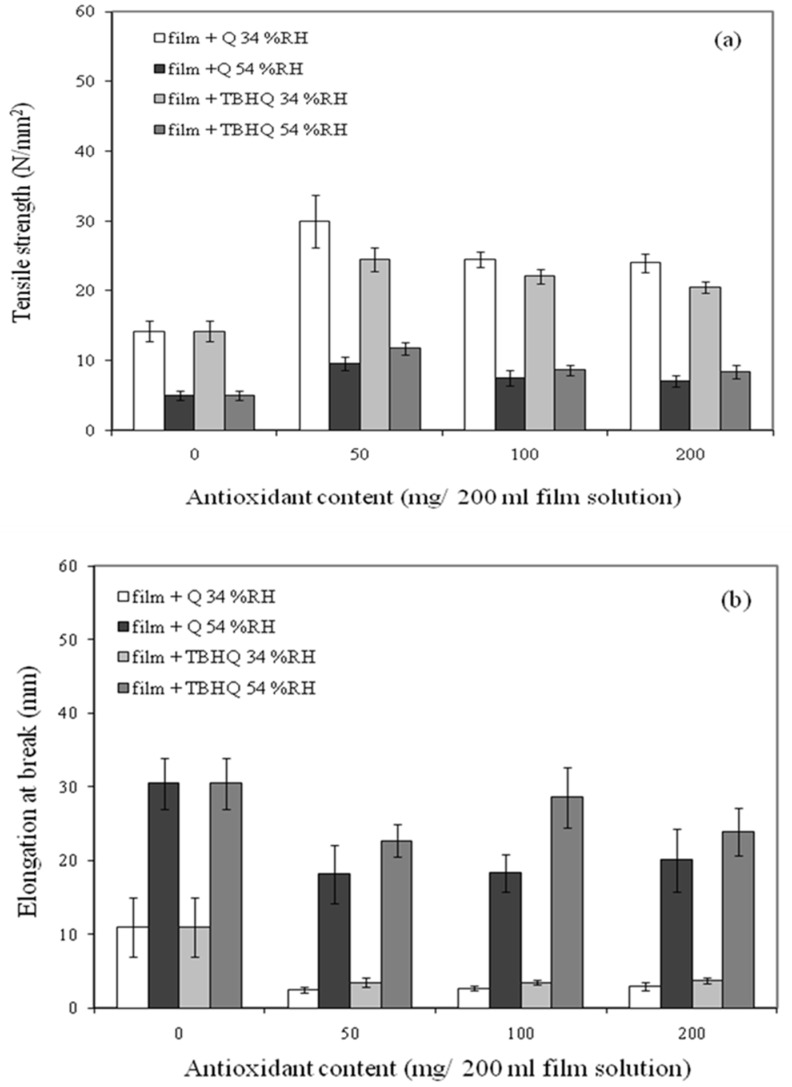
Effect of quercetin and tertiary butylhydroquinone (TBHQ) contents on (**a**) tensile strength and (**b**) elongation at break of cassava starch– carboxymethyl cellulose (CMC films at 34% and 54% RH).

Cassava starch–CMC (7:3) was used in this study to form a film which showed good mechanical properties, as described in a previous study [21]. Antioxidants (quercetin and TBHQ) were added into the film to determine the effect of quercetin and TBHQ concentrations on the mechanical properties of cassava starch–CMC film. Tensile strength (TS) of cassava starch–CMC film with various quercetin and TBHQ concentrations are shown in Figure 1a,b, respectively.

At 34% RH, TS of cassava starch–CMC films with quercetin and TBHQ were higher than the control cassava starch–CMC film. It might be due to a possible interaction between quercetin or TBHQ and cassava starch–CMC, which strengthened the film network. Hydroxyl groups in quercetin and TBHQ possibly acted as hydrogen donors and hydrogen bonds could be formed between quercetin and starch–CMC molecule. Li, et al. [31] described that the larger molecules normally form a stronger network, which increases the energy required to tear the starch film during tensile testing. This result agreed with the TS of fish skin–CMC film incorporated with BHT and α-tocopherol [32]. It related to the elongation at the break of the films, as shown in Figure 1b. Cassava starch–CMC film with quercetin and TBHQ gave a lower elongation at break (EAB) than the control film. However, increasing quercetin or TBHQ content slightly increased the EAB of the film blends. In a comparison of the mechanical properties of the film with quercetin and TBHQ, cassava starch–CMC blended films containing quercetin showed higher tensile strength than the film with TBHQ. Nevertheless, cassava starch–CMC film with TBHQ was more flexible than the film with quercetin. The film containing 50 mg quercetin/200 mL film solution showed the highest TS, and a film containing 100 mg TBHQ/200 mL film solution showed the highest EAB. However, these results were different to those obtained in a previous study with rice flour/cassava starch film containing antioxidants (PG, BHA, BHT) where the type of antioxidant had no effect on mechanical properties of the film [20].

The effect of relative humidity (34% and 54% RH) on the mechanical properties of the film blends was also investigated. All films kept at 54% RH gave higher EAB but lower TS than films at the 34% RH condition, because water worked as a plasticizer by binding with hydroxyl groups (OH) of the starch chain and reduced the intermolecular bonds and increased mobility in polymer chains. This result agreed with Rachtanapun and Wongchaiya [33] who studied the influence of relative humidity on the mechanical properties of the chitosan–methylcellulose film. At 54% RH, increasing quercetin and TBHQ concentrations decreased tensile strength. Increasing TBHQ content increased EAB of cassava starch–CMC films due to the plasticizing effect of increasing absorbed water in film [34,35,36]. On the contrary, the increase of quercetin concentration had no significant effect on the EAB of the film.

### 3.2. Fourier Transform Infrared Spectroscopy (FT-IR) 

**Figure 2:**
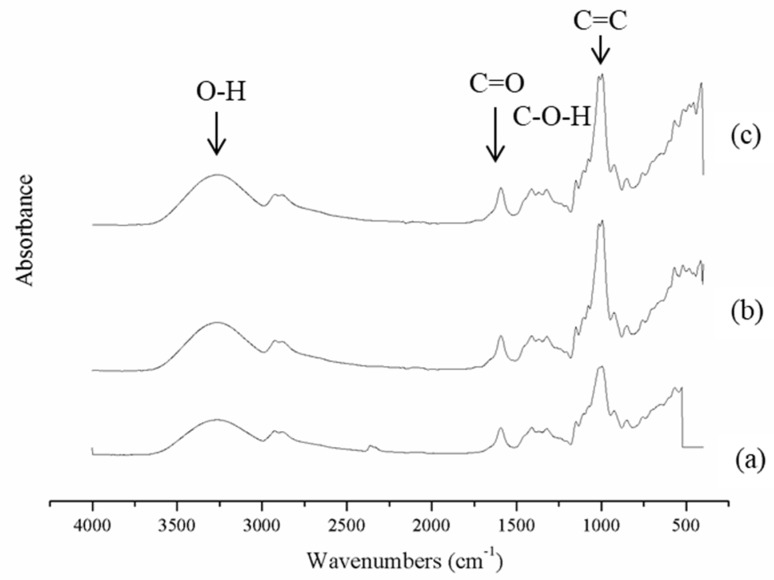
FT-IR spectra of cassava starch–CMC films (**a**) without antioxidant (control), (**b**) with quercetin, and (**c**) with TBHQ.

The FT-IR spectra of control film (without antioxidant) and those incorporated with quercetin and TBHQ are shown in Figure 2. A special mention should be made to the peaks between 3265 and 2926 cm^−1^, matching the stretching vibration of free hydroxyl and –CH band stretching, respectively [37]. Additionally, strong water bands at 1322 cm^−1^, associated with OH in-plane bending, is noticeable in the films incorporated with quercetin and TBHQ. By the addition of antioxidants into cassava starch–CMC film, the O-H band of films shifted to 3263–3260 cm^−1^. The C-OH bending band of cassava starch–CMC film that appeared at 1322 cm^−1^ was shifted to 1326–1323 cm^−1^ with antioxidant addition. The peak at 1100 cm^−1^ related to the glycosidic linkage. The other important change takes place between 1592 and 1100 cm^−1^. Peaks at 1592 cm^−1^ are ascribable to carbon-to-oxygen (C = O) stretching within the carboxylic group of CMC [38]. The slight change in absorption band intensity at 995 cm^−1^ was observed in the composite films when quercetin and TBHQ were incorporated. These results were consistent with the FT-IR spectra of fish gelatin films containing BHT and α-tocopherol [32], and the FT-IR spectra of chitosan films with α-tocopherol [39]. When quercetin and TBHQ were added to the composite films, new peaks at 1369, 1078, and 1015 cm^−1^ appeared that was associated with the stretching of C-O-C, C-O-H bending of carbohydrate chains, and ether bonds [39]. This observation supported that there could be a particular arrangement in the films, due to the interactions of antioxidant polyphenolic compounds with hydroxyl and carboxyl groups of CMC [40]. These results were in agreement with the FT-IR spectra of chitosan film incorporated with green tea extract [27] and the study of physicochemical interaction between chitosan and catechin by Zhang and Kosaraju [41], who found that the peak of the carboxyl group of the chitosan decreased when incorporated with catechin. Similar findings were also reported by Curcio et al. [42] in the formation of covalent bonds between gallic acid–chitosan and catechin–chitosan.

From FT-IR, it is evident that the addition of quercetin and TBHQ could form hydrogen bonding and covalent bonding, and thus engaged the functional group of cassava starch–CMC matrix, and subsequently lowered the free hydrogen group which can form hydrophilic bonding with water [27]. 

### 3.3. X-ray Diffraction Patterns

**Figure 3:**
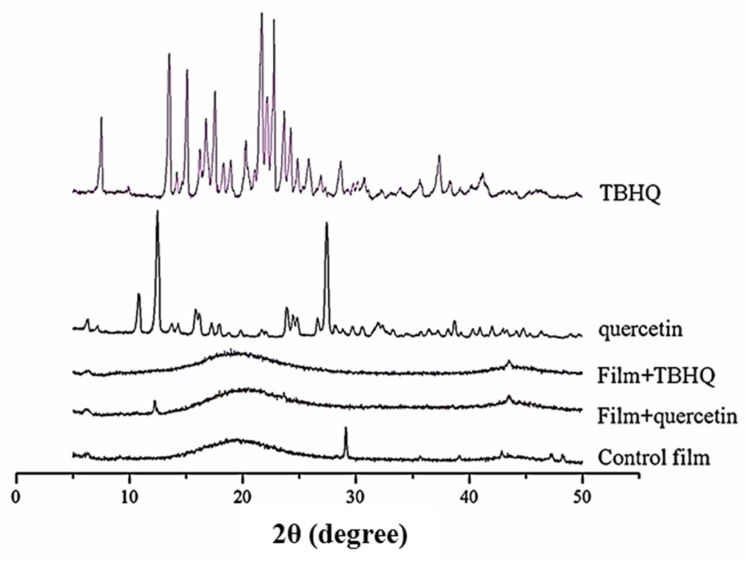
XRD diffractograms of quercetin, TBHQ, control cassava starch–CMC film, and cassava starch–CMC films with quercetin and TBHQ.

The diffractogram obtained for the control film (Figure 3) showed a characteristic pattern reported in the literature [43]; the peaks correspond to amylopectin (pseudo-crystalline) located at 2θ = 16°−19°, due to the CMC–starch interactions. There is a sharp peak located at 2θ = 28°, and a peak located in the region of 2θ = 7°–8°, which are a pattern of CMC [43]. 

However, the pseudo-crystalline and sharp peaks at 2θ = 28° of control film were suppressed when quercetin and TBHQ were added into the composite film (Figure 3). The crystalline peaks of the composite films decreased because the added quercetin and TBHQ blocked the rearrangement of starch–CMC crystallization in composite films [44]. A new broad amorphous peak was observed, demonstrating an interaction between these components [45]. At 2θ = 12°, a new sharp peak occurred in composite films containing quercetin. It represented the crystalline structures of added antioxidants. 

### 3.4. Thermal Properties of the Composite Films

**Figure 4:**
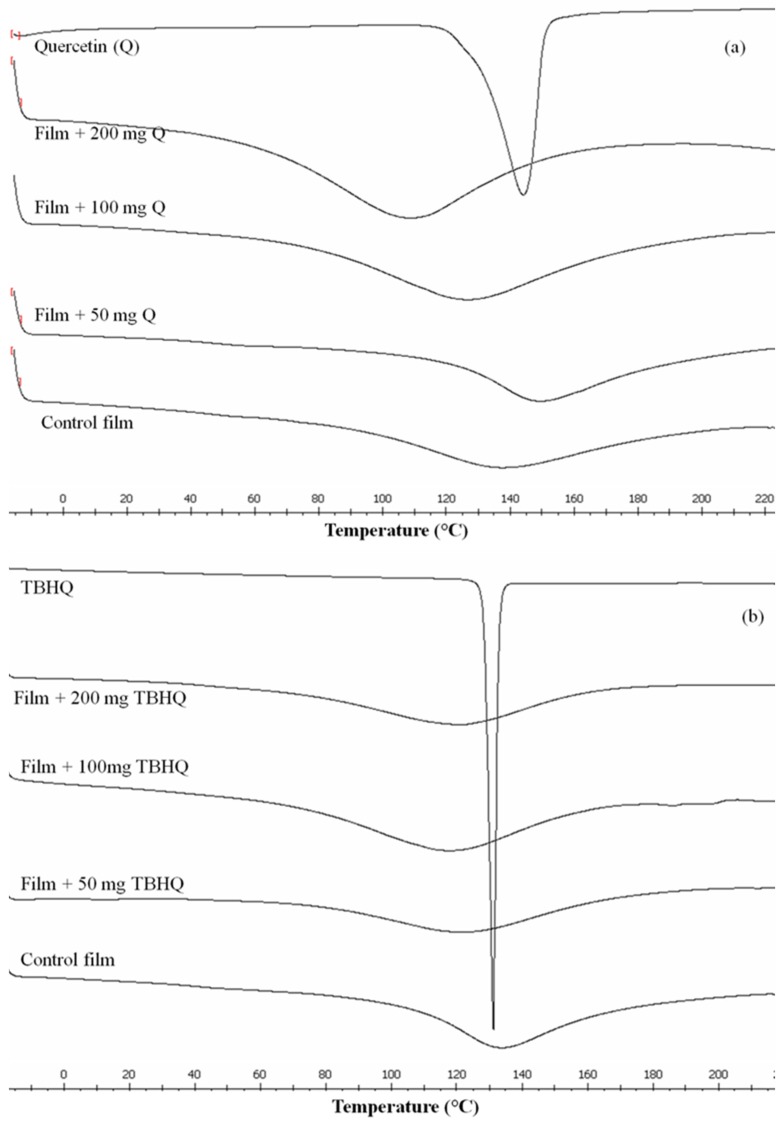
Differential Scanning Calorimetry (DSC) thermograms of cassava starch–CMC films with (**a**) quercetin and (**b**) TBHQ.

The melting temperature (T_m_) and heat of fusion (ΔH) of cassava starch–CMC films, with and without quercetin and TBHQ, are presented in Table 1. Thermograms of the composite films with quercetin and TBHQ showed a single sharp endothermic peak (Figure 4), which indicated homogeneity of the films. This endothermic peak was related to the melting of crystalline starch and CMC domains [46]. This result agreed with DSC thermograms of corn starch–CMC films [43] and soluble starch–CMC films [47]. 

The melting temperatures (T_m_) of cassava starch–CMC composite films with quercetin and TBHQ were lower than the T_m_ of cassava starch–CMC films, except for the film with 50 mg quercetin. According to Arvanitoyannis, et al. [47], polyols interact with starch and CMC polymers, favoring hydrogen bonding formation and decreasing the interactions between polymer chains. This behavior leads to a lower melting temperature. The T_m_ of composite films with 50 mg quercetin shifted to higher temperatures due to the interaction between the film matrix and quercetin [38]. The area under the endothermic peak represented the heat of fusion of the films 39–40, which also improved with increasing quercetin concentrations in cassava starch composite films. It is due to the interaction between the quercetin and film matrix which needs more energy to break bonds [39]. This result related to the mechanical property (highest TS) of the film with 50 mg of quercetin.

On the other hand, ΔH of cassava starch–CMC films with TBHQ was lower than ΔH of control film. This is because the incorporation of TBHQ into the film matrix decreases the intermolecular force between starch–CMC chains, and partly decreases the crystallinity of cassava starch–CMC, resulting in a decrease in the degree of crystallinity in composite films [48] as the TBHQ content increased (as shown in the results from XRD). These results related to the XRD results, which indicated that the addition of quercetin and TBHQ blocked the rearrangement of starch–CMC crystallization in composite films. According to Arvanitoyannis, et al. [47], polyols interact with starch and CMC polymers favoring hydrogen bonding formation and decreasing the interactions between polymer chains. This behavior leads to lower interaction energies between polymer chains. These results were related to DSC thermograms of azuki bean starch films with cacao nibs extract [49] and chitosan–MC films with vanillin [50]. 

### 3.5. Water Solubility of Composite Films

**Figure 5:**
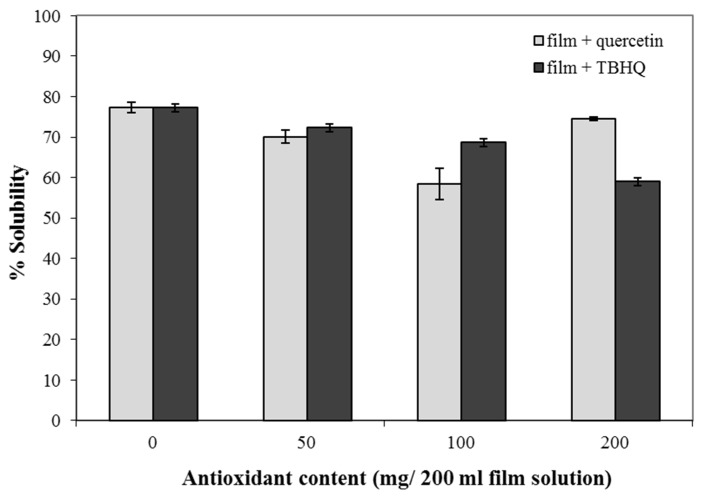
Water solubility of cassava starch–CMC films with quercetin and TBHQ.

The water solubility of cassava starch–CMC composite films was dependent on antioxidant concentrations (Figure 5). The water solubility of the control films was about 78%, and film solubility declined with increasing quercetin and TBHQ concentrations. This result was consistent with the water solubility of cross-linked fish gelatin-chitosan films [51]. Moreover, the solubility of cassava starch–CMC films incorporated with quercetin and TBHQ is also related to the observations of the FT-IR spectra and the mechanical properties of the films, as discussed previously. This indicated that intermolecular interaction [4,47] likely occurred between the antioxidant and starch–CMC in the composite films. The CMC molecules have both positively and negatively charged segments. The two charged segments can join through inter- and intramolecular interactions [52]. The hydroxyl (O–H) group and carboxyl (C = O) of CMC can form strong hydrogen bonds with the hydroxyl groups on the phenolic antioxidant [53], improving the interactions between molecules and the cohesiveness of the biopolymer matrix, and decreasing the water solubility [46]. 

### 3.6. Total Phenolic Content Assay

**Figure 6:**
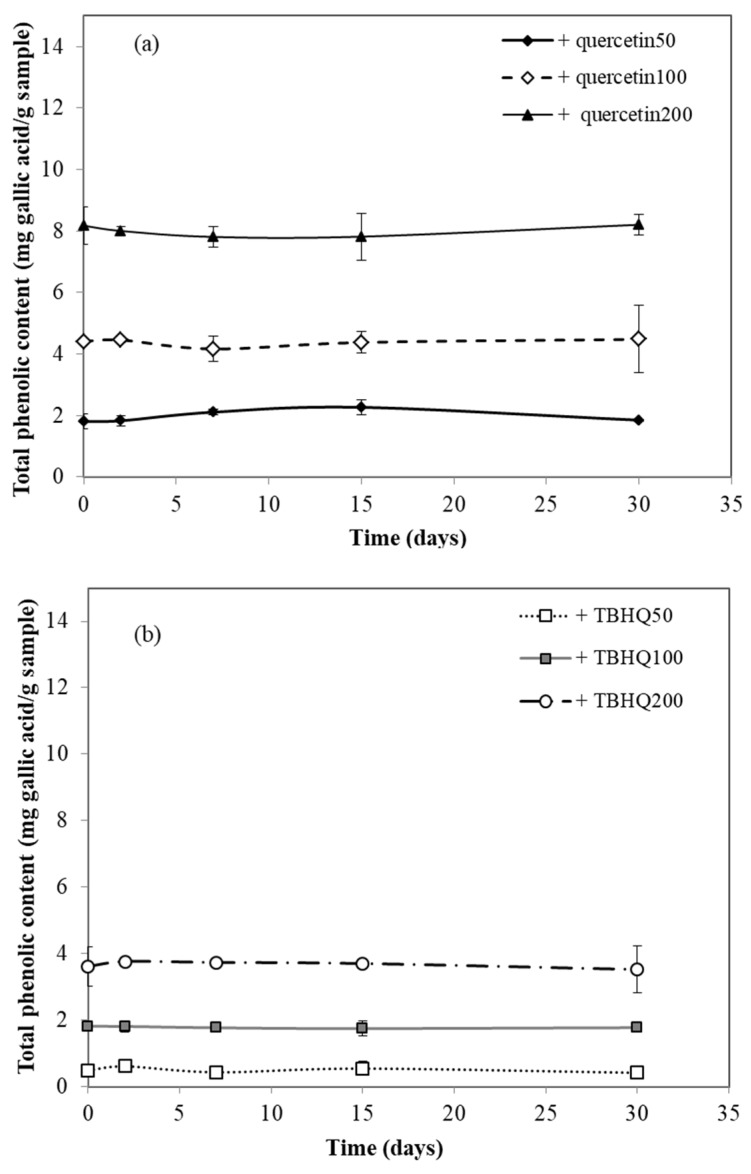
Effect of aging on total phenolic content of cassava starch–CMC films with (**a**) quercetin and (**b**) TBHQ.

The results showed that total phenolic content in the cassava starch–CMC films significantly increased (*p* ≤ 0.05) with increasing quercetin and TBHQ concentration (Figure 6). At the same antioxidant concentration, the total phenolic content of cassava starch–CMC films with quercetin was higher than that of the film with TBHQ, because quercetin has a higher molecular weight (gallic acid equivalent) than TBHQ. Storage time had no effect on the total phenolic content of cassava starch–CMC films.

### 3.7. Determination of Antioxidant Activity in the Composite Films 

The results showed that the DPPH scavenging activity of cassava starch–CMC films was not different (*p* ≥ 0.05) with increased quercetin and TBHQ concentration (data not shown). Storage time had no effect on the DPPH scavenging activity of cassava starch–CMC films. This result agreed with the total phenolic content of the films as described in Section 3.6.

### 3.8. Effect of Antioxidants Incorporated into Cassava Starch–CMC Films on Lard Storage 

**Figure 7:**
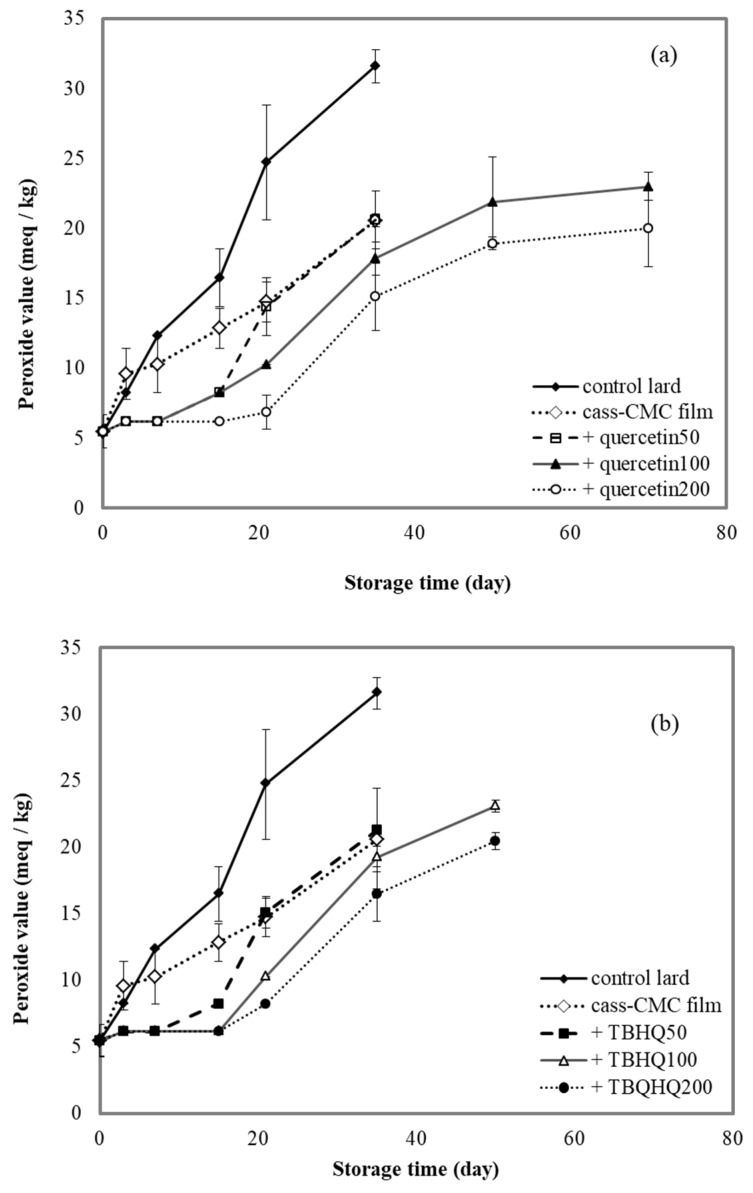
Effect of (**a**) quercetin and (**b**) TBHQ content in cassava starch–CMC films on peroxide value of lard.

To determine the effect of cassava starch–CMC films containing quercetin and TBHQ on the rancidization process, lipid peroxides and lipid aldehydes were monitored during lard storage. Peroxide value (PV) represents primary products of lipid oxidation and is used for the oxidative state determination of lipid-containing foods [29]. As shown in Figure 7, the changes in the PV of the lard were relatively insignificant. This could be explained by the small amounts of polyunsaturated fatty acids contained in lard. The PV of the unpackaged lard (control) increased from 5 to 20 meq/kg (which represented the rancidity of lard) during the 18-day storage period. Even though the PV of the lard packaged with cassava starch–CMC films with and without quercetin and TBHQ also increased, the rate of increase was considerably lower than control. The lard packaged in the film without antioxidants showed lower PV than control, because of lower oxygen permeability through the film. This result agreed with the PV of almond oil in hydroxy propyl methyl cellulose (HPMC) film [54]. Moreover, during the first three days, the PV increase of lard packaged in cassava starch–CMC films containing quercetin and TBHQ was insignificant compared to control and lard packaged in a film without antioxidant, proving that sustained release of the quercetin and TBHQ from the cassava-CMC films inhibited early lipid oxidation [29]. The increase of quercetin and TBHQ content in the films extended the shelf-life of the lard from 18 days to 70 and 50 days, respectively. 

These results showed that cassava starch–CMC films containing quercetin and TBHQ can be used as an active packaging for the postponement of lard oxidation. 

### 3.9. Effect of Antioxidants Incorporated into Cassava Starch–CMC Films on Discoloration of Pork 

**Figure 8:**
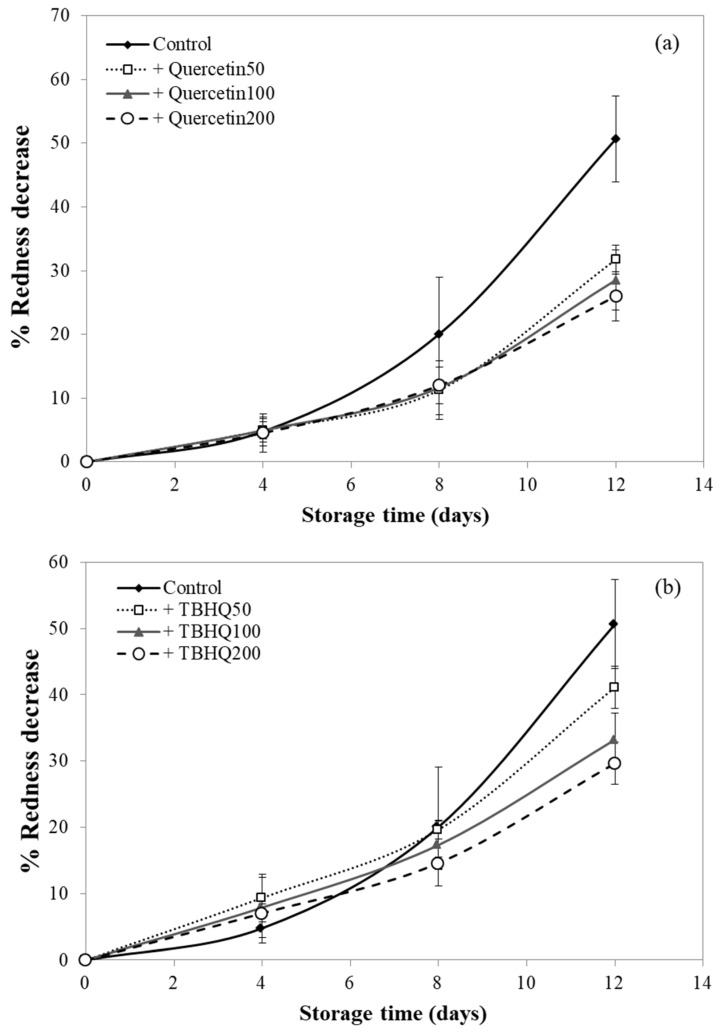
Redness decrease (%) of pork uncovered (control) and covered with cassava starch–CMC film containing (**a**) quercetin and (**b**) TBHQ.

In this study, pork samples were covered with cassava starch–CMC composite films containing various concentrations of quercetin and TBHQ, and then the change of color within 12 days was measured. In order to compare the decrease of redness, % redness decrease was calculated as shown in Figure 8. The pork samples covered with a film containing quercetin and TBHQ had higher (*p* < 0.05) redness than uncovered pork (control) after eight days of storage. After eight days of storage, the redness of uncovered pork decreased rapidly and reduced by more than 50%, whereas the redness of pork covered with the quercetin- and TBHQ-films showed lower redness reduction than control. Redness of pork covered with TBHQ films slightly decreased (less than 20% redness decrease) after 12 days. This indicated that quercetin and TBHQ addition in cassava starch–CMC film gave good results with regard to preventing overall discoloration (Figure 8). These results were consistent with the retardation of pork oxidation using antioxidative plastic film coated with horseradish extract [29], using tea catechin impregnated PVA-starch film on red meat [55], and the color loss reduction in pork loin coated with an alginate-based edible coating containing rosemary and oregano essential oils [46].

Therefore, it was not surprising that the studied films inhibited the oxidation of myoglobin in pork. This result confirmed the antioxidative activity of cassava starch–CMC film with quercetin and TBHQ, which can retard the color discoloration as well as delay the oxidation of lard, as described in Section 3.8. 

## 4. Conclusions

Mechanical properties of cassava starch–CMC film were generally affected by the incorporation of quercetin and TBHQ, as well as relative humidity. Cassava starch–CMC film with antioxidants increased tensile strength, but reduced elongation at the break of the films. Increasing quercetin and TBHQ contents decreased tensile strength, but increased elongation at the break of the films. FT-IR spectra represented the intermolecular interactions between cassava starch–CMC film with quercetin and TBHQ by indication of the shifting of the –OH band, carboxylic group, and aromatic ring. The XRD micrographs also confirmed the interactions of the cassava starch–CMC matrix with antioxidants. DSC thermograms established the homogeneity of films containing quercetin and TBHQ. The increase of quercetin and TBHQ contents decreased the water solubility of the films. In application, cassava starch–CMC film containing quercetin and TBHQ can retard the oxidation of lard (35–70 days) and delay the redness discoloration of pork. 

Thus, it seems that the cassava starch–CMC films containing quercetin and TBHQ showed better physical properties than cassava starch–CMC film. The cassava starch–CMC films containing quercetin and TBHQ have the potential to be used as active and biodegradable films for low and intermediate moisture products.

## Figures and Tables

**Table 1:** Melting temperature (T_m_) and heat of fusion (ΔH) of cassava starch–CMC films incorporated with varying concentrations of quercetin (Q) and TBHQ (T).

Films	T_m_ (°C)	ΔH (J/g)
Cassava starch–CMC	128.65 ± 7.25 ^a,b^	154.14 ± 4.11 ^a^
Quercetin	128.88 ± 3.76 ^a,b,c^	151.00 ± 4.66 ^a^
TBHQ	131.19 ± 5.70 ^a^	273.56 ± 9.77 ^c^
+50 mg Quercetin	141.21 ± 6.13 ^e^	158.20 ± 3.38 ^a^
+100 mg Quercetin	116.09 ± 5.29 ^b,c,d^	211.12 ± 8.37 ^b^
+200 mg Quercetin	110.16 ± 5.66 ^b,c,d^	227.88 ± 7.65 ^b^
+50 mg TBHQ	110.09 ± 0.23 ^d^	147.39 ± 5.73 ^a^
+100 mg TBHQ	118.33 ± 2.88 ^c,d^	144.75 ± 3.52 ^a^
+200 mg TBHQ	119.65 ± 0.44 ^c,d^	149.80 ± 1.13 ^a^

Different letters in the same column indicate significant differences between the means obtained in Duncan’s test (*p* < 0.05).
